# RNA-Seq of Chicken Embryo Liver Reveals Transcriptional Pathways Influenced by Egg Formaldehyde Treatment

**DOI:** 10.3390/genes16050471

**Published:** 2025-04-22

**Authors:** Mustafa Özdemir, Ghulam Asghar Sajid, Selma Büyükkılıç Beyzi, Mehmet Kızılaslan, Yunus Arzık, Servet Yalçın, Stephen N. White, Mehmet Ulas Cinar

**Affiliations:** 1 Department of Animal Science, Faculty of Agriculture, Erciyes University, 38280 Kayseri, Türkiye; mustafa.ozdemir@erciyes.edu.tr (M.Ö.); drgasajid@gmail.com (G.A.S.); sbuyukkilic@erciyes.edu.tr (S.B.B.); 2 Department of Animal and Dairy Sciences, University of Wisconsin–Madison, Madison, WI 53706, USA; kizilaslan@wisc.edu; 3 Department of Animal Science, Faculty of Veterinary Medicine, Aksaray University, 68100 Aksaray, Türkiye; yunus.arzik@aksaray.edu.tr; 4 Department of Animal Science, Faculty of Agriculture, Ege University, 35100 İzmir, Türkiye; servet.yalcin@ege.edu.tr; 5 Poultry Microbiological Safety & Processing Research, United States National Poultry Research Center, United States Department of Agriculture-Agricultural Research Service, Athens, GA 30605, USA; stephen.white@usda.gov; 6 Department of Veterinary Microbiology & Pathology, College of Veterinary Medicine, Washington State University, Pullman, WA 99164, USA

**Keywords:** *Gallus gallus*, mRNA expression, transcription factors, fumigation, chick health

## Abstract

Background/Objectives: Hatchery fumigation is recognized as a crucial step to control microbial bloom in the environment, and formaldehyde is one of the most widely used disinfectants to ensure successful hatchability and healthy production. While many of the benefits are thought to be derived from disinfectant properties, it is possible that additional host gene and genetic pathway modulation could contribute to these outcomes. The current study aimed to capture the in ovo transcriptional response of liver tissue to formaldehyde treatment. Methods: Chick embryos were subjected to formaldehyde fumigation treatment for 25 min at 24–25 °C and 75% relative humidity, keeping a control group as untreated. On the 18th day of incubation at 37.8 °C and 58–63% humidity, eggs were broken, and liver tissue was obtained for RNA isolation, cDNA library preparation, and RNA sequencing. Results: Bioinformatics analysis revealed 908 significant differentially expressed genes (DEGs), among which 814 were known genes and 94 were novel genes. A total of 672 DEGs were upregulated, whereas 236 genes were downregulated in response to FA treatment. Of the 94 novel genes, 80 were upregulated. Key DEGs, associated QTLs, and transcription factors were involved in immuno-inflammatory responses, oxidative stress, epigenetic modification, and cellular adaptation-related activities. Further research should focus on biological validation of key DEGs to clarify their roles, pathways, and relationships to FA treatment. Conclusions: Overall, these findings (1) provide critical molecular detail as a first step towards genetic selection to improve formaldehyde treatment response and effectiveness, and (2) provide DEG signatures for FA treatment as a reference against which to compare other interventions to achieve hatchability and production benefits.

## 1. Introduction

Formaldehyde (FA) is found in organisms in two forms. First, endogenously in which the body produces it and second, exogenously (called also environmental and occupational) sourced from outside the biological system [1]. As a biocide, FA is a colorless, strong-smelling chemical widely used in various industrial applications and as a preservative [2]. Its chemical formula is CH_2_O, and it is the simplest form of aldehyde [3]. In addition to FA’s usage in many working activities and indoor workplaces, it has been widely used in the poultry business to clean brooder houses, hatcheries [4], and feed [5], in addition to prolonging the shelf life of chicken meat [6]. Microbiological contamination of eggshells is a major hazard in poultry husbandry during hatching egg production and handling. The internal structures of the egg may become contaminated by microorganisms on the eggshell surface that are able to pass through structural flaws or pores in the shell [7,8,9]. Thus, microbial contamination of hatching eggs is a main concern of poultry producers, as it causes poor hatchability and chick performance [4]. Furthermore, during the hatching period, these infected embryos act as reservoirs for the horizontal transmission of infections. The humidity in the hatching environment encourages the growth of both pathogenic and apathogenic microorganisms such as during chick hatching. FA gas has long been used as a sanitizer and is typically administered to eggs after they are collected [4,10]. Researchers from various nations have been investigating natural biocidal compounds or ultraviolet treatment as alternatives to bleaching agents [9,10,11,12]; however, to date, no improved alternative to FA application has been found in commercial poultry incubation for large-scale egg sanitization.

Both endogen and exogenous FA can modulate inflammatory responses through many mechanisms: (i) inflammasome activation [13], (ii) induction of glycolysis [14] by DNA-protein crosslinks (DPCs), which obstruct transcription and DNA replication, can be brought on by FA [15,16]; (iii) by DNA adduct formation in which chemicals react with DNA bases [17,18]; and (iv) by oxidative stress in which FA treatment can generate reactive oxygen species (ROS), causing oxidative DNA changes [19]. Fortunately, there are enzyme systems that repair most DNA adducts and crosslinks such as Nucleotide Excision Repair (NER) and/or Base Excision Repair (BER) [20,21,22]. Treatment with FA causes many genes to be expressed differently in some systems [23]. Thanks to high-throughput technologies like RNA-seq, it is now possible to analyze changes in gene expression after being exposed to FA in detail. Research has demonstrated that FA modifies the expression of genes related to several biological processes, such as DNA repair, apoptosis, and cell cycle regulation. On the other hand, a few studies are available that investigate the genetic and epigenetic background of FA in living organisms, and most of these studies have been conducted on humans, model organisms, and cell models [3,24]. Most prior transcriptomic studies focused on the respiratory tract, such as nasal epithelium and bronchial epithelial cells [24]. However, little is known about the transcriptional effects of FA treatment in chicks.

The liver is the largest metabolic organ, and it is involved in the metabolism of numerous biologically important compounds, synthesis of essential proteins, host defense against invading pathogens as well as detoxification of exogenous materials [25,26]. Endogenous FA is rapidly metabolized by various aldehyde-metabolizing enzymes in the body without endangering the organism [27]. However, in high concentrations of FA, toxicity arises in the hepatocytes [28] and this metabolic burden can trigger oxidative injury and inflammation in the liver [29].

The optimization of viable day-old chick production in a poultry hatchery is well recognized to involve the implementation of good cleaning and disinfection methods [4,30]. Losses due to microbial contamination of hatching eggs in the poultry industry can run into millions of dollars [4]. As the most powerful disinfectant that can stop and kill most bacteria that come with hatching eggs or other objects, FA is frequently employed in hatcheries [4,30]. While FA is a highly effective and widely used disinfectant, the full range of biological impacts on developing poultry have not been assessed. To the best of our knowledge, no genetics study about the impact of FA on chicken embryos has been published in the literature. To lay the groundwork for future genetic and epigenetic research on FA treatment to chicken embryos, the aim of current study was to uncover the transcriptomic responses in embryo liver tissue to understand the range of mechanisms in observed benefits using RNA-seq and bioinformatics analysis.

## 2. Materials and Methods

### 2.1. Ethics Statement

This study was approved by the Institutional Animal Care and Use Committee of Erciyes University, Kayseri, Türkiye (4 January 2023, #004). This research protocol adhered to the Turkish Council on Animal Experiment guidelines on farm animal facilities (15 February 2014, #28914).

### 2.2. Chicken Embryo Incubation and Tissue Collection

In the current study, Tinted breed chick embryos obtained from Berrin Enez Hathery and Layer Co. (Balıkesir, Türkiye) were used as hatching eggs in two different incubators (Petersime NV, Zulte, Belgium). The eggs were randomly divided into two groups. Half of the eggs were kept as control and FA was not applied. The other half (*n* = 75 eggs) was exposed to FA application by mixing formalin and potassium permanganate (KMnO_4_) to release FA gas. The heat required for the formalin release was generated by combining 35 mL of formalin with 17.5 g of KMnO_4_ [31]. FA concentration of 6 g/m^3^ was used during this process, and the combustion of FA, egg disinfection, and evacuation of the gas took 25 min [32]. No additional disinfection was performed in the incubator during the study. The temperature was kept between 24 and 25 °C and the relative humidity was kept constant at 75% during fumigation. A total of 150 fertilized chicken eggs were incubated at 37.8 °C and 58–63% relative humidity. On the 18th day of incubation, the embryos were obtained by breaking the eggs and were euthanized by the cervical dislocation method, after which liver samples were taken. This time point was selected based on the Hamburger and Hamilton (HH) developmental staging system, which indicates that by day 18 (approximately HH stage 44–45), the embryo has reached a phase where organogenesis and morphological development are largely complete [33]. Eight developed chick embryos (*n* = 4 from each group) were randomly selected and liver samples snap-frozen in liquid nitrogen and then stored at −80 °C until RNA extraction was performed.

### 2.3. Constructing cDNA Library and RNA Sequencing

Total RNA was isolated from the 8 liver tissues using an RNA extraction kit (Nucleogene, Istanbul, Türkiye) according to the manufacturer’s protocols. RNA purity and concentration were assessed using the BioSpec-Nano small-volume UV Spectrophotometer (Shimadzu Corp., Kyoto, Japan). RNA-seq services were provided by BM Labosis Co., Ltd. (Ankara, Türkiye). The quality of total RNA was determined with RNA Integrity Number (RIN) using the Agilent Bioanalyzer 2100 system (Agilent Tech., Santa Clara, CA, USA) before preparation of cDNA library. RINs of the samples were between 7.8 and 10.0. From the total RNA of each sample, 2 µg was used for library preparation according to the protocol described in the TruSeq RNA Sample Preparation Kit v2 Guide (Illumina, San Diego, CA, USA). After cDNA library preparation, adapter dimers were removed to produce good quality sequencing results. The cDNA libraries were loaded on Illumina NextSeq (Illumina, San Diego, CA, USA) sequencing platform to obtain 150 base pairs (bp) paired end reads.

### 2.4. Preprocessing, Alignment, and Differential Expression Analysis

Paired end reads from both groups were preprocessed to assess the initial quality of the sequencing results using FastQC (https://www.bioinformatics.babraham.ac.uk/projects/fastqc, accessed on 16 November 2024). HTStream (https://github.com/s4hts/HTStream, accessed on 17 November 2024) was used to clean the raw data by eliminating low-quality (bp < 50), duplicates, and adapter reads. The quality of the cleaned data was then rechecked using the FastQC and MultiQC (https://multiqc.info, accessed on 20 November 2024) packages in a Linux system. Chicken reference genome data (https://www.ensembl.org/Gallus_gallus/Info/Index, accessed on 1 December 2024) (gallus_gallus_gca016700215v2) was used to construct the genome index and perform genome alignment with each paired end read of the cleaned data from both groups. For gene quantification and obtaining raw read counts, the Salmon package [34] was used in a Linux system. The quality of the raw read counts was assessed by the shrinking method, and the DESeq2 package was deployed for normalization and differential gene expression analysis between the two groups in RStudio (version: 2024.09.1 + 394). For the determination of significantly differentially expressed genes (DEGs), a cutoff criterion of adjusted probability (*p*)-adjusted value < 0.01 and log_2_(fold change) > 1 was used.

The DESeq2 package utilizes a count matrix *K* with rows representing genes and columns representing samples, where each entry *Kij* indicates the number of sequencing reads mapped to a gene in each sample. For each gene, a generalized linear model (GLM) is fitted, assuming the read counts *Kij* follow a negative binomial distribution, also known as a *γ*-Poisson distribution, with mean *μij* and dispersion *αi*. The mean *μij* is expressed as the product of a normalization factor *sij* and a quantity *qij*, which is proportional to the concentration of cDNA fragments from the gene in the sample. Often, a constant *sj* is used for all genes in a sample to account for differences in sequencing depth between samples. To estimate these size factors, DESeq2 employs the median-of-ratios method, a technique used for normalization by accounting sequence differences between library size and RNA sample composition. GLMs with a logarithmic link function, *log*_2_ *qij =* Σ*r xjr βir*, are used, where the design matrix elements *xjr* indicate the treatment status of sample *j*, and the coefficients *βir* represent the gene’s overall expression strength and the log_2_ fold change between FA and control conditions.

### 2.5. Gene Ontology (GO) and Pathway Enrichment Analysis

Gene annotation was carried out using the free version of the GeneXplain tool (https://genexplain.com, accessed on 4 December 2024) to obtain a holistic picture of differentially expressed genes. GO terms with *p*-value < 0.05 were considered significantly annotated. The results of the GO for both up- and down-regulated DEGs were visualized using the ggplot2 (https://ggplot2.tidyverse.org/, accessed on 5 December 2024) package. Two pathway databases KEGG (https://www.genome.jp/kegg/pathway.html, accessed on 5 December 2024) and Reactome (https://reactome.org/, accessed on 5 December 2024) were employed and the results were rendered by using Pathview package (https://pathview.uncc.edu/, accessed on 6 December 2024) using RStudio and programming (version: 2024.09.1 + 394).

### 2.6. Transcription Factor (TF) Analysis

To analyze the unprecedented details of the regulation of DEGs, a computational methodology was adopted, which involved analyzing the TF regulating these DEGs. For each DEG, the respective promoter sequence was extracted, covering the regions 500 bp downstream to 100 bp upstream relative to the transcription start sites, using gene data obtained from Ensemble database (https://www.ensembl.org/Gallus_gallus/Info/Index, accessed on 10 December 2024). Non-significant DEGs were considered as background regions. The JASPAR2024 non-redundant vertebrate data and the match tool of CiiiDER were employed. This tool compares the distribution of the number of TF binding sites in the significant DEGs with the background of non-significant DEGs using the Mann–Whitney U test. The significance of TFs was determined using the Chebyshev inequality formula.

### 2.7. Positional Mapping of DEGs to QTL Regions

To identify potential candidate genes associated with known quantitative trait loci (QTL), a data fusion technique of positional mapping was performed using the chicken animal QTL database v:GRCg7b (https://www.animalgenome.org/cgi-bin/QTLdb/GG/index, accessed on 12 December 2024) [35]. DEGs and their genomic coordinates were retrieved based on the chicken reference genome (https://www.ensembl.org/Gallus_gallus/Info/Index, accessed on 14 December 2024).

The genomic positions of the DEGs were compared against known QTL regions in the chicken QTLdb. A ±1 megabase (Mb) flanking region was applied to each QTL boundary to cover potential regulatory elements and nearby candidate genes. DEGs located within these QTL intervals were further filtered down using a criterion of log_2_ (fold change) > 2 and QTL *p*-value < 0.05. Genomic comparisons and positional mapping were conducted using R scripting (version: 2024.09.1 + 394).

### 2.8. Validation of RNA-Seq with qRT-PCR

Six DEGs were randomly selected. Total RNA was isolated from the 8 liver tissues using a QuickEx Total RNA extraction kit (Nucleogene, İstanbul, Türkiye) according to the manufacturer’s protocols. The purity of the total RNA was assessed using a Biospec-nano spectrophotometer (Shimadzu Corp., Kyoto, Japan). According to the manufacturer’s protocol, we synthesized cDNA using the NucleoGene RNA to cDNA Mix Kit (Nucleogene, İstanbul, Türkiye). The volume of the reaction mixture was 20 μL, with 3 μL of cDNA, 0.5 μL for each primer, 10 μL of SYBR green (NEB, Boston, MA, USA), and 6 μL of RNA-free water. The following qRT-PCR reaction was performed: 95 °C for 1 min; 95 °C for 15 s, annealing temperature for 30 s for 35 cycles; finally, the melting curve collection at 65 to 95 °C. The expression levels were calculated using the 2^−ΔΔCt^ method normalized with Ubiquitin B (*UB*). Primers used were synthesized by Primer3web [36]. Primers for the *HDC*, *FNIP1*, *BCAN*, *OASL*, *GOLGA7*, and *HNRNPKL* genes are listed in Table S1.

## 3. Results

### 3.1. Sequencing Data and Differential Expression Analysis

A total of 16,348,132 clean reads were acquired, ranging from 192,172 to 4,964,539. The clean reads were characterized by the average GC content of 54.38% and more than 97.12% of Q20, while more than 69.74% of clean reads were mapped to the chicken reference genome (Table S2).

**Figure 1:**
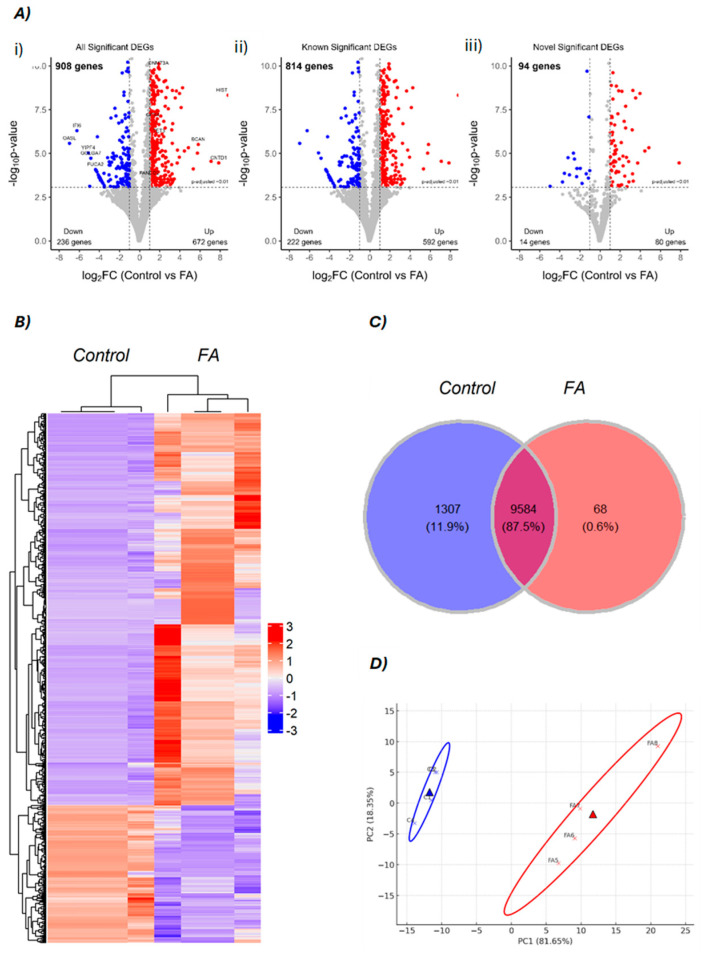
Visualization of differentially expressed genes. (**A**) Volcano plots of differentially expressed genes (DEGs): (**i**) all significant DEGs, (**ii**) known DEGs, and (**iii**) novel DEGs. Red and blue dots indicate upregulated and downregulated genes in FA-treated embryos compared to controls, respectively. (**B**) A cluster heatmap of DEGs, based on z-score between control and FA group, read color shows upregulated genes and blue color shows downregulated genes pattern. (**C**) Venn diagram of overlapping of all genes in this experiment. (**D**) Principal component analysis (PCA) plot illustrates the clustering of groups based on their principal components, control (blue) and the FA group (red).

Our data revealed both significant similarities and differences in differential gene expression and the activity of biological processes under the influence of FA. Differentially expressed gene (DEG) results, including known and novel transcripts as well as their up- and downregulation patterns, are summarized in a schematic overview for clarity and comparison (Figure S1). Differential expression analysis yielded 908 significant DEGs, among which 814 were known genes and 94 were novel genes (Table S3) (Figure 1A). The cluster analysis of all DEGs is mapped in Figure 1B, showing clear differences in gene expression patterns between the control and FA groups. A total of 672 genes were upregulated, while 236 genes were downregulated (Figure 1(Ai)). Among the 814 known genes, 592 were upregulated, while 222 were downregulated (Figure 1(Aii)). Additionally, 80 novel genes were upregulated, and 14 novel genes were downregulated (Figure 1(Aiii)). Among the novel DEGs, the top five most significantly upregulated include LOC107049986 (outer dense fiber protein 3-like), LOC101751691 (D-β-hydroxybutyrate dehydrogenase, mitochondrial-like), LOC121107540 (nuclear receptor domain-containing protein-like), LOC100857834 (pepsin A-like) and LOC121109782 (SUN domain-containing protein 3-like). Conversely, the top five downregulated novel DEGs were LOC107050692 (serine/threonine-protein kinase TAO2-like), LOC426626 (AN1-type zinc finger protein 5-like), LOC101749377 (heterogeneous nuclear ribonucleoprotein L-like), LOC417013 (putative acyl-CoA dehydrogenase AidB-like), and LOC100859737 (tubulin α-1A chain-like). These transcripts represent novel genomic elements responsive to formaldehyde treatment, warranting further investigation for potential regulatory or functional roles during embryonic hepatic response. Analysis of all genes in our experiment shows that 87.5% of genes were co-expressed in both groups, while 1307 genes were uniquely expressed in the control group and 68 genes were uniquely expressed in the FA group (Figure 1C). PCA seemed to separate control and FA into two different groups based on their principal component 1 (Figure 1D).

### 3.2. Identification of Candidate Genes in Quantitative Trait Loci (QTL) Regions

**Figure 2:**
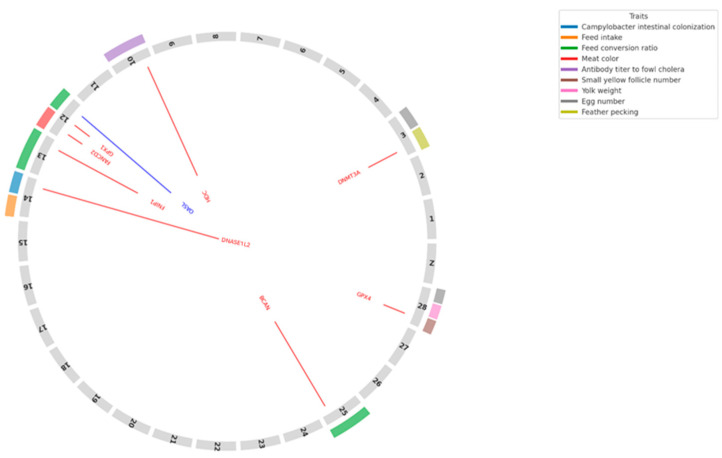
Circular visualization of key differentially expressed genes (DEGs) mapped onto chicken chromosomes alongside relevant QTL (Quantitative Trait Loci) traits. DEGs are shown as colored lines corresponding to their chromosomal positions. Red and blue lines represent upregulated and downregulated genes, respectively, in the FA-treated group compared to controls. Colored blocks on the outer ring denote the chromosomal locations of QTLs associated with economically important traits, including immune response, feed efficiency, and reproductive traits. Trait categories are indicated by color, as defined in the legend box.

We identified nine key DEGs mapped within or near QTL regions associated with important traits in chickens (Table S4) that are illustrated in Figure 2. QTLs for feed conversion ratio (FCR) [37] and feed intake (FI) [38] were detected on chicken chromosomes GGA12, 13, 14, and 25 (Table S4) and the association of FCR and FI with growth traits in chickens is well known [39,40]. The most interesting locus appears to be on GGA12, where three genes were identified within the 3 Mb region associated with FCR (Table S4). Among these, Fanconi anemia group D2 (*FANCD2*) (Table S3) was one of the genes mapped to this locus.

### 3.3. Functional Enrichment Analysis for Differential Expression Between Control vs. FA

**Figure 3:**
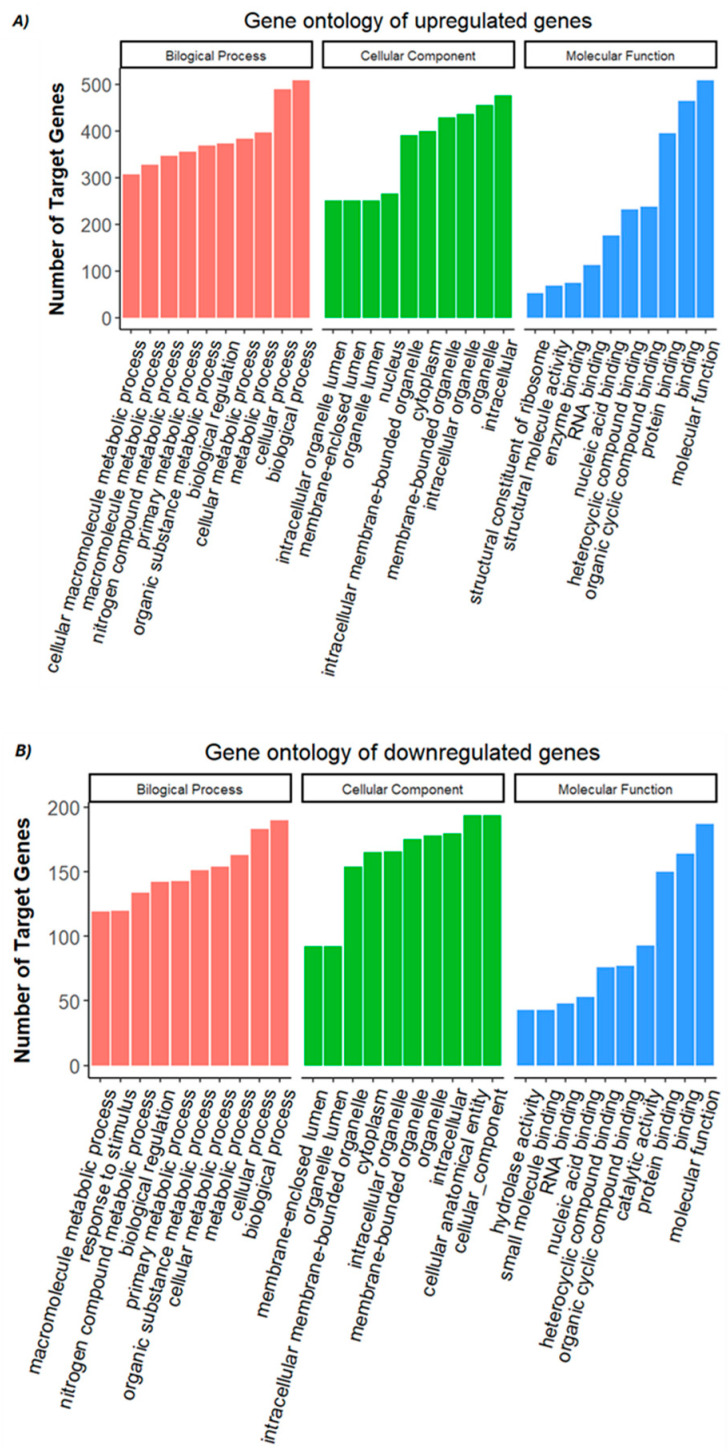
Top ten enriched Gene Ontology (GO) terms for (**A**) upregulated and (**B**) downregulated genes identified in FA-treated versus control embryos. GO terms are categorized into three main domains: biological process (red), cellular component (green), and molecular function (blue). Enrichment analysis was performed based on statistical significance, and the top ten terms in each category were ranked by *p*-value < 0.05.

**Figure 4:**
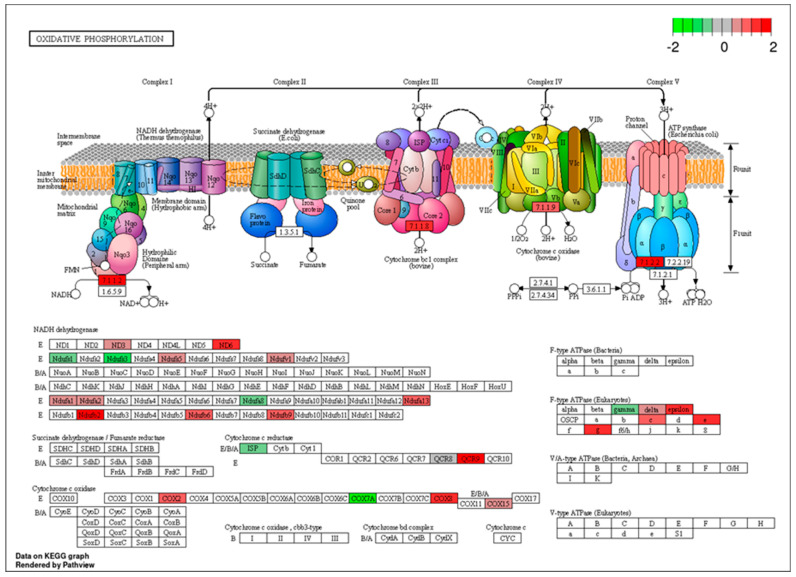
Analysis of oxidative phosphorylation KEGG pathway rendered by Pathview package in R, log_2_ (fold change) differences in DEGs in FA group are represented by colors: red color denotes upregulation and green color denotes downregulation.

Gene ontology (GO) enrichment analysis revealed that the upregulated genes in the FA-treated group were primarily associated with binding-related molecular functions, such as protein binding, organic cyclic compound binding, heterocyclic compound binding, nucleic acid binding, and RNA binding (Figure 3A; Table S5). Enrichment was also observed in categories related to enzyme binding and structural roles, particularly structural molecule activity and ribosomal structural components, indicating a potential increase in translation and protein processing activity. Conversely, the downregulated genes were enriched in GO terms associated with both binding and catalytic functions. These included protein binding, small molecule binding, nucleic acid binding, and RNA binding, as well as catalytic activity and hydrolase activity, suggesting a suppression of certain enzymatic and metabolic processes under FA exposure (Figure 3B; Table S5). KEGG and Reactome pathways were employed on DEGs. KEGG enriched pathways include ribosome, oxidative phosphorylation, drug metabolism involving other enzymes, protein processing in the endoplasmic reticulum, and pyrimidine metabolism. The PathView of oxidative phosphorylation is illustrated in Figure 4. Reactome pathways included nonsense mediated decay (NMD), selenocysteine synthesis, translation and peptide chain elongation, and response of EIF2AK4 (Table S6).

There were several Reactome pathways involved in lipid metabolism and glucose metabolism, including metabolic pathways, PI3K–Akt signaling pathway, glycolysis/gluconeogenesis, and steroid biosynthesis. Upregulated genes were enriched into metabolic pathways and steroid biosynthesis, while downregulated genes were related to metabolic pathways, the PI3K–Akt signaling pathway, and glycolysis/gluconeogenesis. In GO terms, upregulated genes were enriched in the biosynthesis and metabolism of cholesterol and sterol, the metabolism of fatty acids, and cellular lipid catabolism, while downregulated genes were related to lipid metabolism, response to lipid, regulation of lipid metabolism, and carbohydrate metabolism processes.

### 3.4. Transcription Factor (TF) Analysis

A total of 36 significant TFs were identified, among them 23 TF were over-represented, while 13 were under-represented (Table S7). Most of these belong to basic helix loop helix (bHLH), Sp, Kruppel-like factors (KLF), and Zinc finger families of transcription factor. Four TFs cooperation; STAT1::STAT2, ETV5::FIGLA, TFAP4::FLI1, and TAL1::TCF3 were also seen in the regulation of DEGs.

### 3.5. Validation of the Results by qRT-PCR

**Figure 5:**
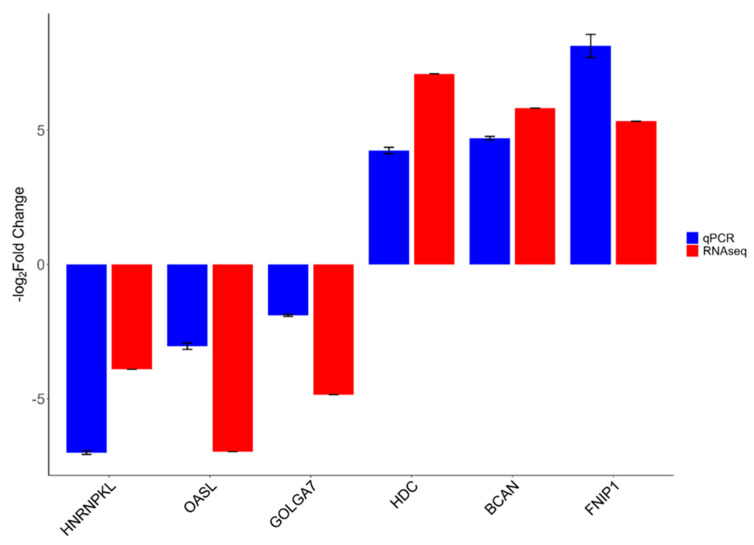
Validation of expression levels of DEGs by qRT-PCR results for control vs. FA, *n* = 3. The expression trends of the six genes (Control vs. FA) as determined by RNA-seq and qRT-PCR were entirely congruent. Fold change represents the ratio of mean expression of the control group to FA administration.

qRT-PCR validation results verified the accuracy of the sequencing data for six randomly selected genes (*HNRNPKL*, *OASL*, *GOLGA7*, *HDC*, *BCAN*, and *FNIP1*) from 908 DEGs (Table S3). The experimental results of these genes were consistent with the sequencing results, indicating that the sequencing results in this study were reliable (Figure 5).

According to the validation results, six genes were similar to the RNA-seq results of the control and FA groups and were consistent with expectations. The direction and magnitude of expression changes observed through RNA-seq were consistently reflected by qRT-PCR despite variations in fold-change magnitudes. Specifically, *HNRNPKL*, *OASL*, and *GOLGA7* were downregulated according to both RNA-seq and qRT-PCR data, with the most substantial downregulation observed in *HNRNPKL*. Conversely, the genes *HDC*, *BCAN*, and *FNIP1* displayed pronounced upregulation, confirmed by both methodologies. Notably, while fold-change values from qRT-PCR and RNA-seq were broadly consistent, a slight difference was observed in the magnitude for *FNIP1*, where qRT-PCR indicated a marginally higher expression level than RNA-seq.

## 4. Discussion

Several genes involved in immune regulation were differentially expressed in response to FA exposure, indicating that the immune system may be a key target of FA-induced transcriptomic changes. Notably, several novel DEGs expressed with high log_2_ fold changes, including LOC107049986 and LOC101751691. These findings suggest robust transcriptional activation in response to FA exposure, which could have significant implications for our understanding of stress adaptation mechanisms. For instance, LOC107049986, annotated as an outer dense fiber protein 3-like gene, may play a role in cytoskeletal or structural reorganization during stress [41], although its precise function remains to be elucidated. Similarly, the mitochondrial dehydrogenase-like gene LOC101751691 could indicate alterations in energy metabolism and redox homeostasis, highlighting the complex interplay between stress responses and metabolic pathways [42]. The marked downregulation of LOC107050692 and other kinase- or zinc-finger-related transcripts suggests that key signaling, or transcriptional regulatory pathways may be suppressed as part of a tightly controlled stress response [43]. Given that these genes are currently uncharacterized in chickens, further functional validation through gene editing or knockdown studies will be crucial to unravel their exact roles and relevance to liver metabolism and development under FA stress conditions.

Among the upregulated genes Dnase1l2 deoxyribonuclease 1-like 2 (*DNASE1L2*) (Table S3) has a role in inflammatory processes associated with increased gene expression during transcriptional activation of inflammatory cytokines [44]. This suggests that *DNASE1L2* may play a critical role in regulating the immune response by participating in DNA destruction during bacterial infection-induced inflammation. In addition, *DNASE1L2* could potentially play a role in degrading damaged DNA fragments [45]. Upregulation of the Histidine decarboxylase (*HDC*) (Table S3) indicates an inflammatory and immunomodulatory processes of the immune system by increasing histamine production; this gene appears to be an important regulator in response to environmental stimuli, especially given that histamine-secreting bacteria in the gut can influence lung inflammation [46]. The potential implications of these mechanisms for inflammation and animal health management should be evaluated in future research. Among the downregulated genes, we found that 2′-5′-oligoadenylate synthetase like (*OASL*) (Table S3) gene may indicate attenuation of the immune response and suppression of interferon signaling [47]. Considering the critical role of the *OASL* gene in antiviral immune responses reported by Ranaware et al. [48], this decrease in gene expression may increase the susceptibility of the embryonic immune system to infections, especially highly pathogenic viral infections such as avian influenza. Similarly, Del Vesco et al. [49] reported that silencing the *OASL* gene significantly reduced the expression of genes involved in the interferon response and apoptosis and resulted in increased levels of viral RNA in cells infected with Newcastle disease virus. Suppression of the *OASL* gene may contribute to increased viral load by reducing the effectiveness of antiviral defense mechanisms. Interferon-α inducible protein 6 (*IFI6*) (Table S3) is an important interferon-stimulated gene [50]. As accomplished by Wan et al. [51], the *IFI6* gene was shown to support antiviral responses in avian reovirus (ARV) infection, and its high expression suppressed ARV replication. Conversely, diminished *IFI6* expression has been documented to facilitate viral replication by reducing its regulatory influence on immunological factors. In addition, the experiment carried out by Yu et al. [52] showed that *IFI6* gene expression in chickens infected with infectious bursal disease virus was markedly increased in the early and middle stages of infection and that this increase supported antiviral immune responses. These findings support that the *IFI6* gene may play a critical role in immune regulation during infection.

The regulation of these genes indicates that FA application significantly impacts inflammatory processes, and this effect may be related to both the regulation and resolution of inflammation. Our findings significantly contribute to understanding the impact of FA on the expression of inflammation-related genes. In particular, these findings may guide the development of new approaches to combat infections or stress-induced inflammatory processes in poultry. The liver plays a pivotal role in appetite regulation [53], and dysfunction in hepatic metabolism may significantly influence appetite and, consequently, traits such as FCR and FI in chickens [54]. Fanconi anemia complementation group D2 (FANCD2) has been reported to provide protection against formaldehyde-induced DNA damage by participating in DNA repair mechanisms [55], which may explain its upregulation in our study. Oxidative stress is a major consequence of FA exposure, often resulting from ROS accumulation and disruption of the cellular redox balance. Understanding the transcriptomic response to FA treatment helps in elucidating the molecular mechanisms underlying effects from administration. FA treatment potentially triggers a cascade of molecular responses depending on submission type and dose, primarily involving immuno-inflammatory responses, oxidative stress, epigenetic modification, and cellular adaptation related activities [56]. A key aspect of FA exposure is its ability to generate ROS, ultimately altering cellular redox balance and triggering cellular response [56,57]. In the current study, the observed dysregulation of glutathione peroxidase 1 (GPX1) and glutathione peroxidase 4 (GPX4) (Table S3) indicated the involvement of the glutathione redox system in mitigating oxidative stress. Notably, key components of this system, including glutathione-disulfide reductase (GSR), glutathione synthetase (GSS), GPX1, glutathione peroxidase 3 (GPX3) and GPX4 are integral to glutathione redox system in chickens [58]. Glutathione peroxidases (GPXs) play a crucial role in neutralizing oxidative stress by detoxifying the hydrogen peroxide radicals into water or alcohol, ultimately protecting cellular damage [59]. Additionally, FA treatment upregulates tumor necrosis factor α (TNFα), a pro-inflammatory cytokine known to induces ROS production. Increased ROS levels, in turn, triggers mitogen-activated protein kinase kinase kinase 7 (MAP3K7), which upregulate the expression of COX2 gene (Table S3), a key mediator of inflammatory responses [60,61]. These findings align with our results and highlight the intricate interplay between oxidative stress and inflammation in response to FA treatment. In addition to its effects on immune and oxidative pathways, FA is a well-known genotoxic agent that induces DNA damage, particularly through the formation of DNA–protein crosslinks (DPCs). In the current study, the upregulation of Fanconi anemia complementation group D2 (FANCD2) was identified (Table S3), which is reported as a crosslink repair gene [62]. A key mechanism of FA-induced genotoxicity is the formation of DPCs, which block essential cellular processes such as DNA replication and transcription. The accumulation of DPCs contributes to genetic instability and increases the risk of mutation. In response to such damage, cells activate the repair mechanisms or initiate the pathways to remove the damaged DNA, ensuring genomic integrity [63]. In addition to these genetic effects, FA treatment is potentially involved in non-genetic changes such as DNA methylation and histone modifications [24]. In DNA methylation, DNA methyltransferases (DNMTs) enzymatically transfer a methyl group from donor S-adenosyl methionine (SAM) to the fifth carbon of the cytosine base, converting it into 5-methylcytosine (5mC) [64]. Upregulation of *DNMT3a* (Table S3) provides a clue that DNA methylation was altered in the current study. However, it is reported that the expression of DNMT3a and DNMT3b was downregulated in 16HBE cells after a long-term low-dose FA treatment. These contradictory results might be due to species, cell type, developmental stage, and FA treatment differences. In addition to genotoxicity, FA exposure has been shown to influence epigenetic mechanisms such as DNA methylation and histone modifications, which can alter gene expression without changing the underlying DNA sequence. In addition to this, DNMT3a potentially modulates the broader cellular response to manage FA mediated effects, as enhanced expression was noted in the prefrontal cortex after a 2 h acute restraint stress [65]. To support this argument, ten-eleven translocation 3 (TET3) was upregulated in the current study (Table S3), and this enzyme converts 5mC into 5-hydroxymethylcytosine (5hmC), 5-formylcytosine (5fC), ultimately into 5-carboxylcytosine (5caC) through its hydroxylase activity. Furthermore, decarboxylation of 5caC helps in DNA demethylation and maintains the cellular integrity [66]. These findings underscore the molecular and cellular response of the liver cells of chicken eggs after a FA fumigation treatment, specifically epigenetic and oxidative stress responses that might have broader implications in case of predisposition to disease conditions.

TFs play a crucial role in regulating genes by binding to specific regions of DNA, called transcription factor binding sites (TFBSs), typically located between the +500 bp to −100 bp to promoter region. In transcriptional studies, activation of specific TFs provides an extra layer of understanding of transcriptional response in any condition [67]. TFs of the current study are aligned well with the activated molecular processes in response to FA such as NR2F2, NFYB, RORα, and SNAI2 regulate the oxidative stress [68,69,70]. E2F2 was dysregulated in response to FA and DMPT treatment in nasal cavity transitional cell epithelium of rats; E2F1 and E2F2 protect the genomic stability with the interaction of γH2AX DNA repair factor in response to oxidative DNA modification [71,72]. HOXA1 dysregulation correlated with chemotherapy response, and it was targeted by miR-10 in lung cells, suppressing the carcinogenic effects of FA observed in some systems [73,74]. These TFs provide further evidence of activation of FA induced epigenetic modification and cellular adaptation related cellular activities.

RNA-seq can detect genes and discover transcripts with high sensitivity and a large detection range [75]. The RNA-seq analysis conducted in this study provided a comprehensive understanding of the biological processes affected by FA exposure, particularly those related to immune response, oxidative stress, and epigenetic regulation. Identifying key differentially expressed genes, such as *GPX1*, *GPX4*, *OASL*, *IFI6*, *DNMT3a*, and *TET3*, enabled a clearer interpretation of the molecular alterations induced by FA during embryonic development. In addition to elucidating these molecular effects, our findings may also support practical applications, including refining disinfection strategies and developing biomarkers to monitor embryonic stress responses. Several genes from DEGs list of the current study have known roles in poultry research, particularly in growth, muscle development, disease resistance, and egg production. Genes like *TLR4* [76,77], *IGFBP5* [78], *MEF2C* [79], *IGF2* [80], and *VTG1* [81] have been considered in genetic selection programs for improving poultry production efficiency. However, to utilize them in selective breeding programs to mitigate the negative impacts of FA fumigation, further validation of these DEGs is necessary, as recommended in the conclusion section.

## 5. Conclusions

FA is widely used in poultry hatcheries for egg disinfection purposes and poses a toxicity concern. Chicken serves as a source of animal protein for humans and a great animal model for scientific research; however, there is a clear lack of reports on the regulation of gene expression in chicken liver tissue exposed to FA. Our work is the first to compare chicken embryo gene expression of FA vs. control groups. These data illuminate pathways underlying FA treatment benefits, both through disinfection and potentially also through separate mechanisms. Differential expression analysis identified a total of 908 significant differentially expressed genes, comprising 814 previously known genes and 94 novel genes. A total of 672 genes were upregulated, whereas 236 genes were downregulated. Of the 94 novel genes, 80 were upregulated while 14 novel genes were downregulated. This study revealed significant changes in genes associated with immune responses, oxidative stress, and epigenetic regulation in embryo liver tissue. The findings of this study provide a foundational basis of molecular mechanisms and responses to FA fumigation of hatching eggs. Further research should be focused on biological validation of key DEGs to clarify their roles in immune response, oxidative stress, and epigenetic modification pathways and how they relate to FA treatment. Once validated, these results could lead to improved genetic selection programs and might suggest additional interventions to improve poultry and egg production.

## Data Availability

Sequence datasets were submitted to the NCBI (National Center for Biotechnology Information) Gene Expression Omnibus (GEO) and are available under the accession number GSE287311.

## Supplementary Materials

The following supporting information can be downloaded at: https://www.mdpi.com/article/10.3390/genes16050471/s1, Figure S1: Summary of DEGs; Table S1: Primers for validation of DEGs by qRT-PCR; Table S2: Summary of sequencing reads mapping to the reference genome and quality parameters; Table S3: List of all significantly differentially expressed genes (DEGs); Table S4: List of candidate DEGs positionally mapped with QTL regions; Table S5: GO annotation classes overrepresented with upregulated DEGs; Table S6: List of functional pathways associated with DEGs; Table S7: List of significant transcription factors (TFs). Refs. [37,38,82,83,84,85,86,87,88] are cited in Supplementary Materials.
