# Supplementary material for: RNA-Seq of Chicken Embryo Liver Reveals Transcriptional Pathways Influenced by Egg Formaldehyde Treatment
Source: Genes (Basel). 2025 Apr 22;16(5):471. doi: 10.3390/genes16050471 (PMC12111442; doi:10.3390/genes16050471)
Supplement: Supplementary file 1 [file genes-16-00471-s001.zip › genes-3558843-Figure S1.pdf]

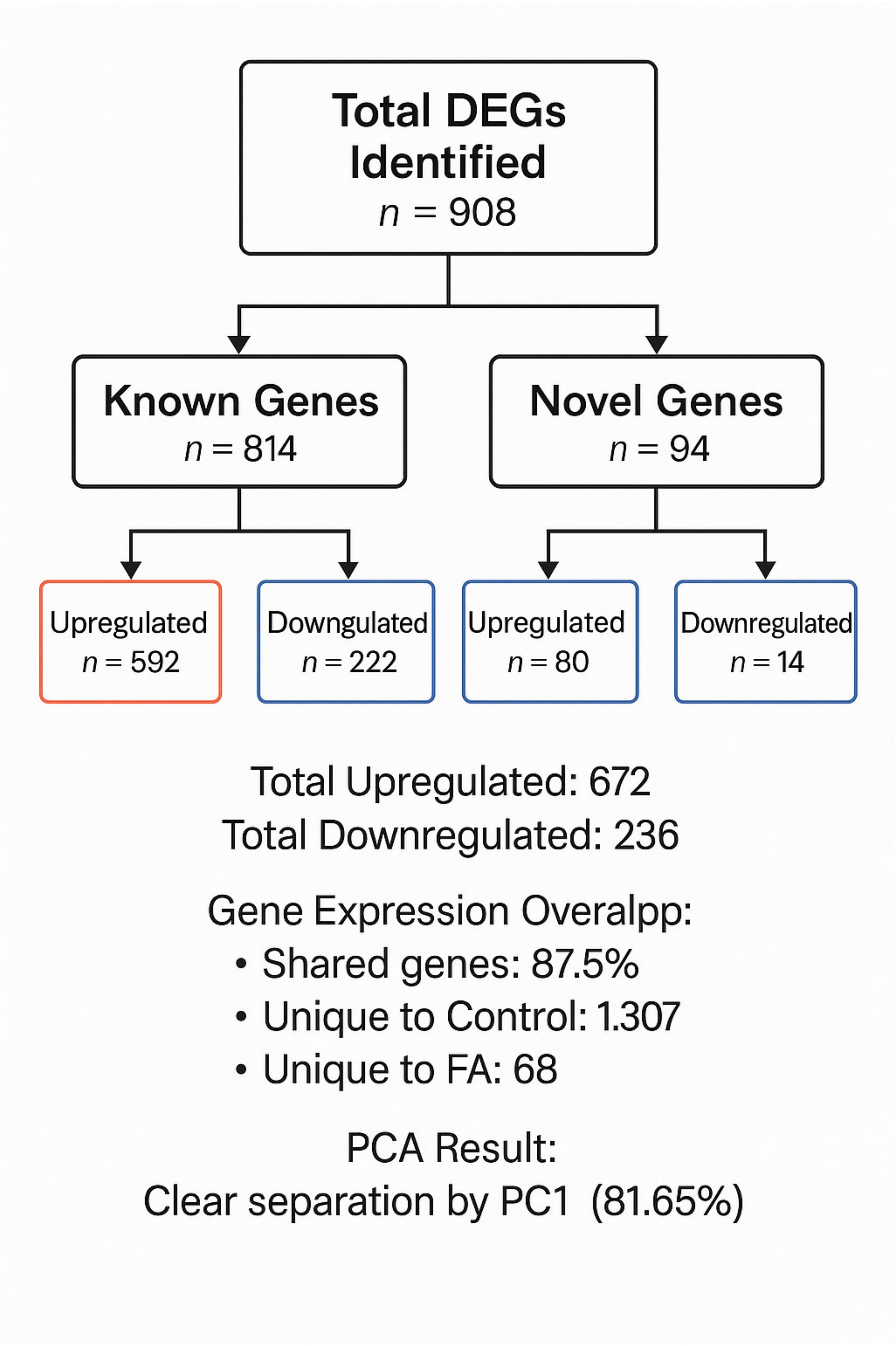

Figure S1. Summary of DEGs.
