# Supplementary material for: RNA-Seq of Chicken Embryo Liver Reveals Transcriptional Pathways Influenced by Egg Formaldehyde Treatment
Source: Genes (Basel). 2025 Apr 22;16(5):471. doi: 10.3390/genes16050471 (PMC12111442; doi:10.3390/genes16050471)
Supplement: Supplementary file 1 [file genes-16-00471-s001.zip › Supplementary Table S1.pdf]

**Supplementary Table S1.** Primers for validation of DEGs by qRT-PCR.

| Gene Name | Gene accession number | Forward primer (5′ → 3′) | Reverse primer (5′ → 3′) | Amplicon size (bp) |
|---|---|---|---|---|
| Histidine Decarboxylase (*HDC*) | ENSGALE00010175302 | CCGGAAGAGGTGATGATGCT | CTGAAGACCACACTGAATGCT | 102 |
| Folliculin Interacting Protein 1(*FNIP1*) | ENSGALE00010159490 | ACGCTGGAGAAAGGAGAAGT | CCTCAGACTCCTTTGGTAGCA | 91 |
| Brevican (*BCAN*) | ENSGALE00010297026 | GCTGCAGGAAAATATCAAGCC | TCGCGACTCAATCTCCACTT | 114 |
| Oligoadenylate synthase-like (*OASL*) | ENSGALE00010289120 | CCTGTGAAGGTGCAAGTGA | ACCACTCCTTCTCTATCTCCC | 106 |
| Golgin A7 (*GOLGA7*) | ENSGALE00010182498 | GGCAGCAGTTTGAAGAGACT | ACAGGCCAGACACCCTTCAA | 98 |
| Heterogeneous nuclear ribonucleoprotein (*KHNRNPK*) | ENSGALE00010002049 | GCCCAGCCTTATGATCCTAAT | AGCCTCCTCTTCCACGCATT | 118 |
| Ubiquitin (*UB*) | M11100.1 | CTCAGGGGTGGCTATTAGTTGT | TAGAAAGACTACAGTGCAACACAC | 83 |
